# Structure validation in chemical crystallography

**DOI:** 10.1107/S090744490804362X

**Published:** 2009-01-20

**Authors:** Anthony L. Spek

**Affiliations:** a Utrecht University, Bijvoet Center for Biomolecular Research, Padualaan 8, 3584 CH Utrecht, The Netherlands

**Keywords:** validation, *checkCIF*, *PLATON*

## Abstract

Automated structure validation was introduced in chemical crystallography about 12 years ago as a tool to assist practitioners with the exponential growth in crystal structure analyses. Validation has since evolved into an easy-to-use *checkCIF*/*PLATON* web-based IUCr service. The result of a crystal structure determination has to be supplied as a CIF-formatted computer-readable file. The checking software tests the data in the CIF for completeness, quality and consistency. In addition, the reported structure is checked for incomplete analysis, errors in the analysis and relevant issues to be verified. A validation report is generated in the form of a list of ALERTS on the issues to be corrected, checked or commented on. Structure validation has largely eliminated obvious problems with structure reports published in IUCr journals, such as refinement in a space group of too low symmetry. This paper reports on the current status of structure validation and possible future extensions.

## Abstract 2

This paper reports on the current status of structure validation in chemical crystallography.

## 1. Introduction

In the late 1960s, only 40 years ago, a routine small-molecule crystal structure determination in the setting of a well equipped crystallography laboratory would take several months. The bottlenecks were the data-collection, structure-solution and structure-refinement stages. Since then, data collection has advanced from a time-consuming film-based and serial detector-based technique to the current area detector-based systems, thus speeding up this stage by at least an order of magnitude. Modern CCD detector-based systems can easily collect 1000 small-molecule data sets in a year. The currently available direct methods for structure solution have essentially solved the long-standing phase problem in small-molecule crystallography given crystals of sufficient quality. Easy-to-use structure-determination software is now widely available and often comes with the data-collection hardware. The computing power needed for data processing, structure solution and refinement, once expensive and a monopoly of the University Computer Centre, is nowadays ubiquitous, cheap and fast on the personal computer platform. Therefore, given a routine structure determination, it is now quite possible to collect diffraction data, solve and refine the structure and send off a structure report for publication in *Acta Crystallographica Section E* within a day. This development is clearly demonstrated by the growth in the number of small-molecule structures that are published each year. This number has increased exponentially over the past 40 years from about 1000 in 1967 to over 35 000 in 2007. It should be noted that this last figure is a lower bound of the actual number of small-molecule structure determinations that are carried out each year. It is likely that a similar number of studies never reach the literature. The publication of a crystal structure as part of a research paper is still a time-consuming activity and remains a bottleneck, often together with the problems of obtaining publication-quality crystals.

Nowadays, the majority of small-molecule crystal structures are determined to ‘confirm’ the outcome of synthetic chemical work. The confirmation of a newly prepared compound by a crystal structure is generally a requirement for the publication of the associated chemistry in major chemical journals. *Seeing is believing*. Crystallography is in this sense often used as an analytical tool. However, there is a problem. The number of experienced crystallographers dedicated to single-crystal studies has certainly not increased in proportion to the number of reported studies. Many single-crystal structure analyses are currently carried out by non-experts using the available black-box software. Often, for understandable reasons, such investigators lack sufficient experience to avoid the many possible pitfalls, such as an incorrect atom-type assignment, that may be obvious to an expert. In the past, all unusual aspects of a structure analysis were supposed to be discussed in a publication with sufficient detail for both the reader and referee to make their own judgment about a claimed result. Nowadays, crystallography is considered by many chemical journals as routine and the crystallographic information is, at best, supplied in a footnote or as supplementary material with very limited details, if any, given in the published text. The chances are therefore high that papers are accepted for publication without crystallographic referees ever having looked at the supporting material. Unfortunately, the number of experienced crystallographic referees has decreased dramatically. As a result, the literature and databases, such as the Cambridge Structural Database (CSD; Allen, 2002 ▶), include obviously incorrect structures associated with formally refereed papers.

About 12 years ago (Linden, 2007 ▶), a crystal structure-validation project was started in the context of the journals of the International Union of Crystallography in order to address the refereeing issue and the time-consuming work that went into the checking of the supplied data for completeness and consistency. Its initial implementation was used to evaluate papers submitted to *Acta Crystallographica Section C*. At that time, it was already a requirement of the journal that the crystallo­graphic data had to be provided in the computer-readable CIF format (Hall *et al.*, 1991 ▶). The submission of electronic data files allowed the validation software to per­form a number of quality and validity checks and to create a report in the form of ALERTS on issues to be addressed by authors and referees. Soon afterwards, further validation tests on structural issues were added. These tests are incorporated as part of the structure-analysis tools that are available in the *PLATON* package (Spek, 2003 ▶; Müller *et al.*, 2006 ▶).

The official IUCr structure-validation suite (*checkCIF*/*PLATON*) is currently available as an IUCr web service (http://journals.iucr.org/services/cif/checking/checkfull.html). Its use is required for every small-molecule crystal structure submitted for publication in the IUCr journals. Many major journals currently have similar requirements, as stated in their Notes for Authors. This paper reports on the current status of the IUCr validation project.

## 2. Structure validation

Structure validation addresses three simple but important questions:

(i) Is the reported information complete?
(ii) What is the quality of the analysis?
(iii) Is the structure correct?

The answer to the first question involves the use of a computerized checklist. The answers to the other questions are obviously less straightforward. The quality of a single-crystal study can be classified into one of four classes.

Class I consists of high-quality structure determinations that were carried out using data collected from a near-perfect crystal and under optimal experimental conditions. This will generally be data collection at a sufficiently low temperature and to a sufficiently high resolution. Such conditions are not always attainable. Inherently poor-quality crystals, disorder or a phase transition can be reasons why this goal cannot be reached.
Class II structures are good structures that were determined under routine conditions or with experimental restrictions that are sufficient for the purpose of their study but not necessarily to the highest attainable quality. This class includes structures from data collected at room temperature or with high-pressure cells.
Class III structures are poor structures that are essentially correct as far as the associated chemistry is concerned but for various reasons have limited accuracy. Reasons can be poor crystals, incomplete or weak and noisy diffraction data. Severe disorder that is difficult to model can be another reason.
Class IV structures are incorrect. Important examples are those in which some of the element-type assignments are wrong or models with too few or too many H atoms. The impact of an incorrect published structure may be disastrous for research that builds on it. Examples include attempts to synthesize complex natural products on the basis of an incorrectly reported crystal structure (for an example, see Li, Burgett *et al.*, 2001 ▶; Li, Jeong *et al.*, 2001 ▶).

Ideally, most issues reported by the validation software should already have been corrected at an early stage of the analysis and thus should never appear in published structures. Correction at the publication stage may be laborious or even impossible for unique crystalline samples. Clearly, structure validation is particularly important for addressing Class IV structures. Class III structures may be useful to direct further research, but are generally not suitable for publication unless supported by an in-depth analysis. Crystallographic journals will aim at Class I structures, while noncrystallographic referees of chemical journals may even be satisfied with Class III structures. Validation should avoid having Class IV structures ever appear in print.

The holy grail of structure validation is a tool that unequivocally assigns one of the above four quality classes to a given structure report. This would be performed on the basis of the application of objective criteria to the supplied structural and experimental data. The currently available IUCr tool, *checkCIF*/*PLATON*, is in this sense still far from that ideal. Instead, a list of ALERTS is produced that are classified according to their level of seriousness. These should be addressed by the investigator and those remaining evaluated by experts. The validation criteria currently in use are in many cases empirical and based on experience and tradition rather than based on science. Some criteria have changed over time. There is an obvious trade-off between being too critical, leading to too many false ALERTS, and being less sensitive and thus missing multiple weak indications of a serious problem. Eventually, a scientifically sound underpinning of the validation criteria will be sought.

Automated structure validation as it is today has its origin in the definition of the CIF standard for the exchange and archival of structural and experimental data (Hall *et al.*, 1991 ▶). CIF became ‘the standard’ in small-molecule crystallography with its adoption by the widely used *SHELXL* refinement-software package (Sheldrick, 2008 ▶). *Acta Crystallographica Section C* made CIF the required data-submission format for publication and it is currently the only way to submit a structural report to *Acta Crystallographica Sections C* and *E*.

Initially, software was developed to check the completeness of the supplied data, its consistency and its validity. It was soon realised that the availability of coordinate data also made it possible to base geometry and other calculations on these data. Examples are the detection of solvent-accessible voids in a structure that were missed by the investigators and the search for missed higher symmetry. This can be achieved by the use of readily available tools in the *PLATON* package (Spek, 2003 ▶).

Validation issues are subdivided into four categories:

(i) Missing or inconsistent data.
(ii) Indicators that the structure model may be wrong or deficient.
(iii) Indicators that the quality of the results of the study may be low.
(iv) Cosmetic improvements, queries and suggestions.

The validation software assigns one of four severity levels (A, B, C and G) to reported issues. Level A ALERTS usually indicate that corrective action is imperative or there has to be a scientifically acceptable explanation for the case at hand. Level G ALERTS concern issues that may be correct but should be checked. They can still point to serious problems that could not be analyzed in detail on the basis of the available data. Currently, about 400 validation tests have been implemented. Most tests result in a one-line ALERT message. Each test is associated with some documentation explaining the problem with possible options to address them.

## 3. Validation of the diffraction data

Most problems with and questions related to a structure report can be resolved just using the data available in the CIF. However, reflection data in computer-readable format will sometimes be needed in borderline cases for a detailed analysis of issues such as the correct symmetry description. Some problems, such as missed or ignored twinning as an explanation for an unsatisfactory refinement result, may only show up in an analysis of the reflection data. The submission of reflection data as a structure-factor file (*F*_o_/*F*_c_ data in CIF format) is required for a structural publication in *Acta Crystallographica*. This allows automatic checking for missed twinning. Absolute structure assignments are generally inferred from the value of the Flack parameter that is reported in the CIF (Flack, 1983 ▶). This value can be erroneous (Flack *et al.*, 2006 ▶) and lead to false conclusions about enantiopurity. The availability of the reflection file allows software to check the reported value independently. This is performed by a com­parison of the value of the reported Flack parameter with the value of the Hooft parameter (Hooft *et al.*, 2008 ▶), which is calculated from the Bijvoet differences. The availability of reflection data also allows an independent structure determination and inspection of difference density Fourier maps for special features such as missing or incorrectly positioned H atoms. Unfortunately, the referees of chemical journals have no easy access to the reflection data since there is no deposition requirement by non-IUCr journals. Consequently, those primary data are also not archived. The Cambridge Structural database does not archive reflection data either.

The validation of *F*_o_/*F*_c_ data is available with the standalone *PLATON*/*VALIDATION* software (http://www.cryst.chem.uu.nl), and will be available shortly through the IUCr *checkCIF*/*PLATON* web service. Validation utilizing the reflection data is currently implemented for papers submitted to *Acta Crystallographica Sections C* and *E*.

## 4. Examples

This section reviews a number of published structure reports that have been shown to be erroneous and for which a formal correction has appeared in the literature. There are many more (largely undocumented) examples of troublesome reports. Any analysis of the data for a subset of structures taken from the nearly 500 000 structures in the CSD will show outliers. Most of these outliers point under close inspection to unresolved problems or errors of some sort rather than being of scientific interest. Unfortunately, in most cases the primary data (reflection data) are unavailable for a proper objective and definitive analysis.

### 4.1. Missed symmetry

The assignment of the correct space group of a structure to one of the possible 230 space groups can at times be problem­atic. The effective space group cannot always be assigned uniquely at the start of the structure analysis on the basis of the observed systematic absences alone. Often, preliminary structure solution only succeeds in a space group that turns out to be a subgroup of the real one. In fact, difficult structures can often only be solved in the lowest symmetry space group *P*1, leaving the transformation to the correct space group to be performed afterwards. Unfortunately, many examples in the literature (see Marsh & Spek, 2001 ▶) show that this goal is not always achieved. The required transformation is not always trivial. Software that suggests the real symmetry and performs the associated transformation is readily available (*e.g. PLATON*/*ADDSYM*), but is not always part of the refinement software suite being used. Some missed symmetry cases are relatively harmless in that this error does not seriously affect the structure and its interpretation (*e.g.* wrong Laue group), such as Example 1 below. On the other hand, overlooking an inversion centre is generally serious. This last problem can be hidden when structure refinement is performed by using constraints and restraints to secure the stability of the least-squares refinement. There are many borderline cases for which the reflection data are needed for a definitive space-group assignment.

#### 4.1.1. Missed symmetry: Example 1

**Figure 1:**
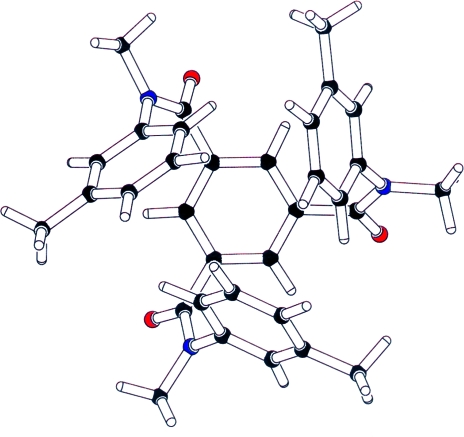
The asymmetric unit of a structure that was originally reported in the orthorhombic space group *Pbca*. The molecular threefold axis is obvious from this projection. The real space group, *Pa* 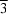, has the molecular threefold axis coinciding with a crystallographic threefold axis.

Fig. 1 ▶ illustrates an example of a structure that was published with one crystallo­graphically independent molecule in the orthorhombic space group *Pbca* (Azumaya *et al.*, 1995 ▶). A program that displays a structure perpendicular to the main molecular plane by default will immediately show that this molecule has at least pseudo-threefold axial symmetry. Such an axis may or may not coincide with a crystallographic axis. The existence of crystallographic threefold symmetry was shown to be the case by Herbstein (1999 ▶). The correct cubic space-group assignment, *Pa* 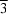, would have been indicated by the current validation software.

#### 4.1.2. Missed symmetry: Example 2

**Figure 2:**
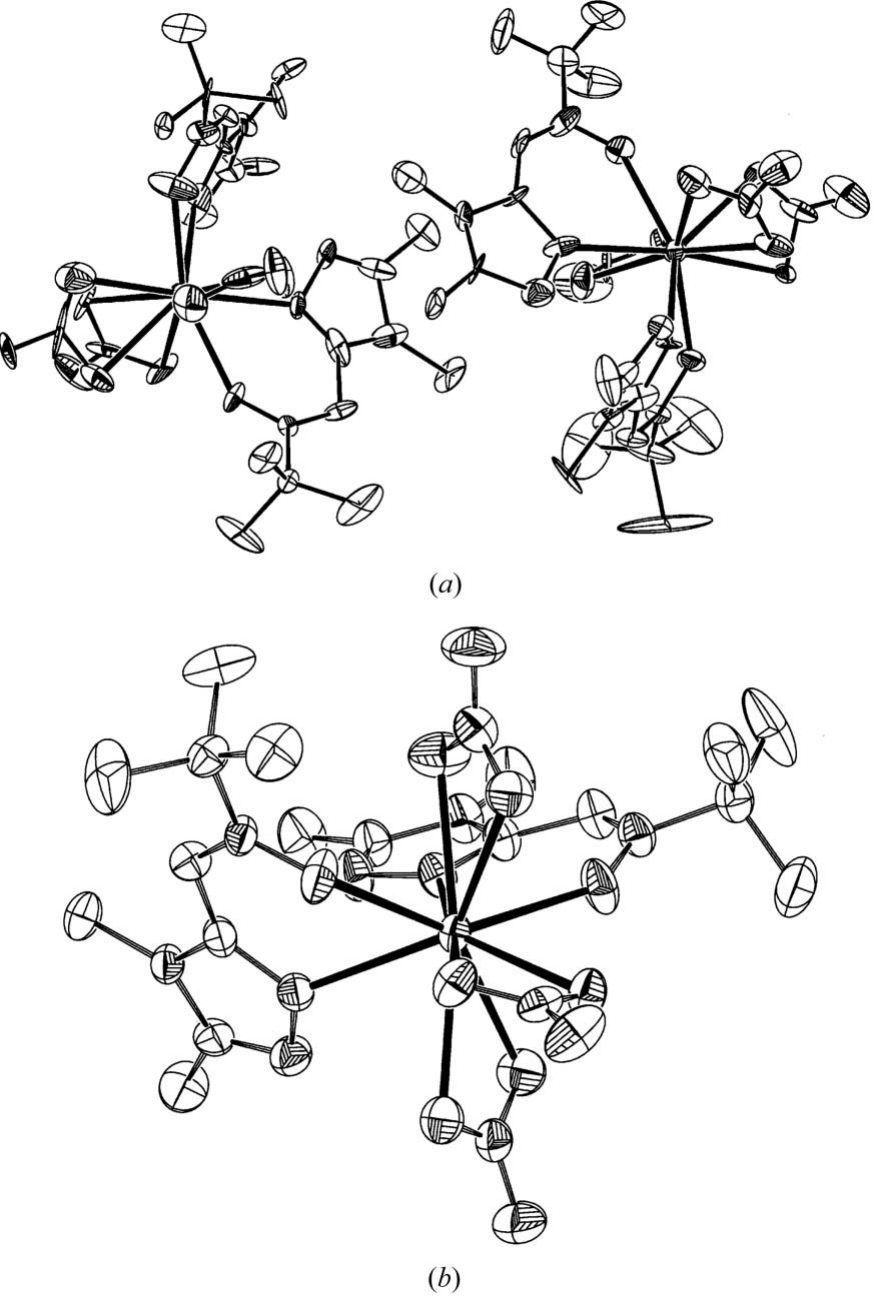
(*a*) Displacement-ellipsoid illustration for a praseodymium complex that was wrongly refined with *Z*′ = 2 in space group *P*1. Note that the largest components of the ellipsoids of ‘inversion’-related atom pairs are perpendicular. (*b*) Displacement-ellipsoid illustration of the same complex refined with *Z*′ = 1 in space group *P* 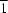. Note in (*a*) the inversion centre in the centre of the figure that relates the two molecules.

Fig. 2 ▶(*a*) illustrates the dramatic effect of the solution and erroneous refinement of a centrosymmetric structure in a noncentrosymmetric space group (Kahn *et al.*, 2000*a* ▶). Even just the published displacement ellipsoid plot of this structure, which has been refined in space group *P*1, should have aroused serious suspicion with the referees of the paper about the quality and correctness of the structure. This structure would have been a perfect candidate for the ‘*ORTEP* of the Year’ award (Harlow, 1996 ▶). It was only on the basis of a suggestion from a reader of the journal that this structure was re-refined in the centro­symmetric space group *P* 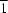. The correctly refined structure, shown in Fig. 2 ▶(*b*), clearly looks quite normal (Kahn *et al.*, 2000*b* ▶). Thus, what might have looked like a structure report based on very poor data turned out to be a good-quality structure after all. In this context, it is interesting that the detailed discussions in the original paper about the unusual differences in bond distances turned out in hindsight to be based on incorrectly interpreted refinement artifacts. The *checkCIF*/*PLATON* validation report (using the downloadable CIF) for the original *P*1 structure cites the space-group problem and numerous other issues.

### 4.2. Missing or incorrectly placed H atoms

Missing H atoms or too many H atoms in a reported molecular structure may have a significant impact on the interpretation of the chemistry or the nature of the compound. H atoms are often introduced to the model at calculated positions without checking whether there is significant electron density at that location or are erroneously left out. Hydroxyl moieties generally have their H atom on a cone and pointing to a hydrogen-bond acceptor in the structure. Exceptions are rare and are generally the consequence of misplaced H-atom positioning, incomplete structures or wrong atom-type assignment.

#### 4.2.1. Missing H atoms

**Figure 3:**
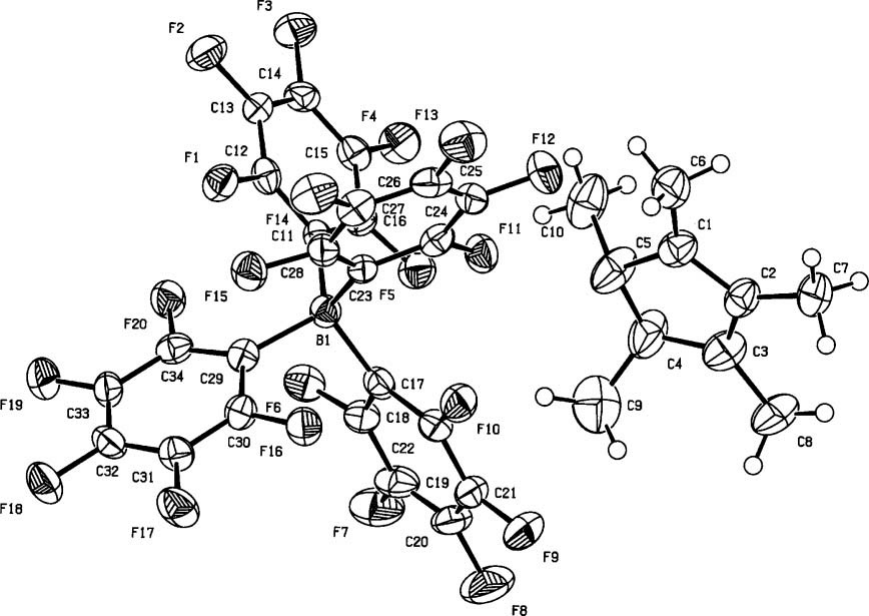
The reported structure with missing H atoms. Atoms C4 and C5 are clearly out of the plane of the five-membered ring and suggest *sp* ^3^ hybridization. In fact, H atoms need to be added at atoms C4 and C5.

Fig. 3 ▶ shows a structure that was published as a synthetic breakthrough with the title *The stable pentacyclopentadienyl cation* (Lambert *et al.*, 2002 ▶). Interesting chemistry building upon this result was envisioned. ‘Packing effects’ were offered as an explanation for the unusual nonplanarity of two substituents on the five-membered ring. It was rapidly shown by Otto *et al.* (2002 ▶) that the reported structure obviously needed two additional H atoms at *sp* ^3^ positions on the five-membered ring and that the reported structure was actually the less interesting pentamethylcyclopentenyl cation. Given the availability of reflection data, it was easy to verify the presence of the two additional H atoms in a difference density map.

#### 4.2.2. Wrongly placed H atom

**Figure 4:**
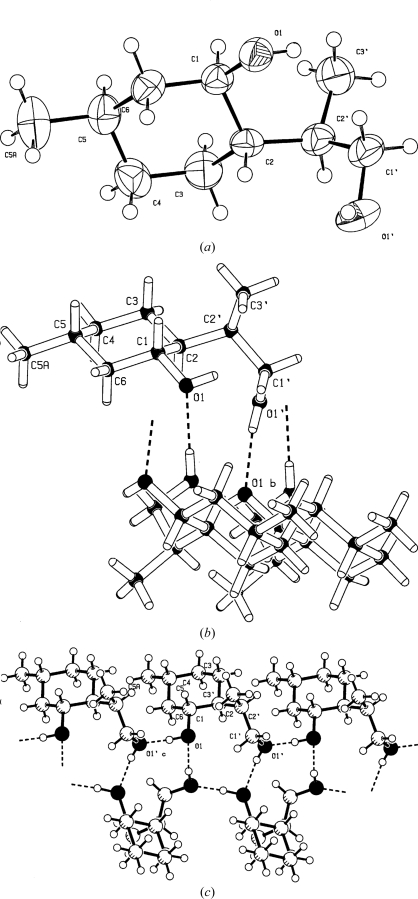
Example of a misoriented hydroxyl moiety with no hydrogen-bond contacts. (*a*) Isolated molecule. The H atom on atom O1 is incorrectly positioned. (*b*) The original hydrogen-bond network with the ‘zombie’ H atom. (*c*) The correct hydrogen-bond network.

Fig. 4 ▶(*a*) shows a structure with an incorrectly positioned hydroxyl H atom (Körner *et al.*, 2000*a* ▶). The problem cannot be seen in a published single-molecule *ORTEP* illustration. What is needed is an analysis of the intermolecular interactions. Fig. 4 ▶(*b*) illustrates the problem that was detected in a retrospective validation run. The correct hydrogen-bond network shown in Fig. 4 ▶(*c*) makes more sense (Körner *et al.*, 2000*b* ▶). Contoured difference electron-density maps can be very helpful in analyzing this type of problem. A misplaced H atom will show up as a negative density peak in its false location and the correct location will appear as a positive peak.

### 4.3. Incorrect atom-type assignments

The result of a crystal structure determination is not always the expected one. In such cases, atom-type assignments may be biased by preconceived ideas and assumptions. Linden (2007 ▶) reports several cases in which the reported chemical species is nearly certain to be wrong. Structures published as possessing —C=N—H groups may sometimes have resulted from a misinterpretation of —C=O groups. Zhong *et al.* (2007 ▶, 2008 ▶) report the retraction of a coordination complex with a missing H atom on an N atom and a central Sn^IV^ atom that is most likely the cation of a lanthanide(III) coordination complex.

Below are two further examples in which the reported chemistry was incorrect.

#### 4.3.1. Withdrawn misinterpreted structure

**Figure 5:**
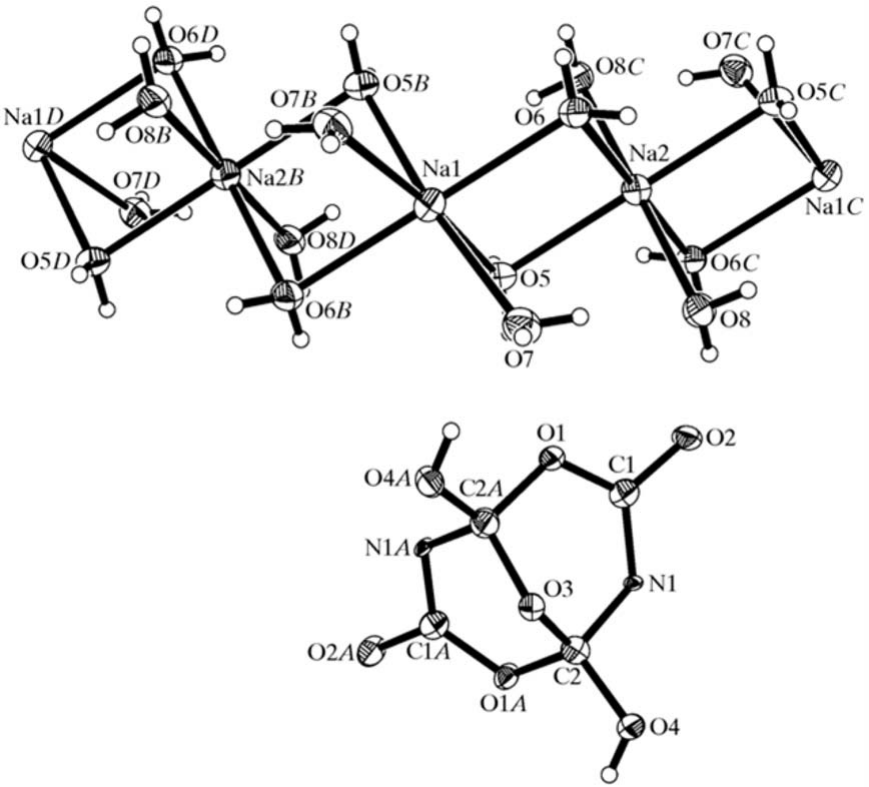
A misinterpreted and retracted structure that turned out to be that of the mineral borax. The atoms labelled N should be oxygen and those marked C should be boron. Figure taken from Fang *et al.* (2008 ▶).

Fig. 5 ▶ is an example of a structure report (Fang *et al.*, 2007 ▶) on a ‘novel heterocyclic’ compound, crystals of which were obviously obtained unexpectedly from a reaction mixture. A reader (an *Acta Crystallographica Section C* Co-editor) recognized this structure as being at least isomorphous with the well known structure of the mineral borax. Closer inspection revealed that the two compounds were indeed identical. The displacement ellipsoids of the N and C atoms clearly suggested that they should be interpreted as the atom types O and B, respectively. Hirshfeld (1976 ▶) rigid-bond test ALERTS sent out similar signals. The structure report was subsequently retracted (Fang *et al.*, 2008 ▶).

#### 4.3.2. Charge-balance problem

**Figure 6:**
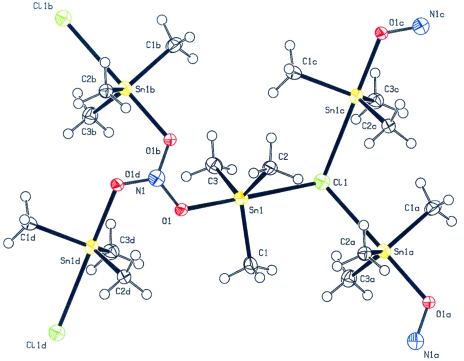
Erroneous network structure with a charge-balance problem. The displacement ellipsoid of N atom N1 is relatively large. The nitrate anion was reinterpreted as a carbonate anion.

Fig. 6 ▶ shows a published network structure (Sadiq-ur-Rehman *et al.*, 2007 ▶) that was obtained unexpectedly. It is not clear from the reaction conditions where the NO_3_^−^ anion in the proposed structure is supposed to come from. In addition, there is also a charge-balance problem that was obviously overlooked by both the authors and the referees of the paper. An anion with a −2 charge is needed. The same authors (Sadiq-ur-Rehman *et al.*, 2008 ▶) have now corrected the structure in view of the charge-balance problem. The NO_3_^−^ anion was replaced by CO_3_^2−^, as suggested by the unusual size of the displacement ellipsoid of N in the NO_3_^−^ version. Generally, such a change of atom type would result in significantly better displacement parameters and refinement results. In this case, no significant improvement was observed. Interestingly, the revised report also does not mention that the reflection data were from a merohedrally twinned crystal. Part of the reason for this might be that the current CIF file definition (and for that reason software such as *SHELXL*) does not yet offer a standard means of recording twinning in a CIF. The twinning correction that was correctly applied was detected as part of the validation of the reflection file. On the other hand, the general implementation of a check for charge balance is a challenging validation issue.

## 5. Evaluation and discussion

An analysis of the ALERTS generated for the 35 760 entries added to the CSD from 2006 and early 2007 indicates that validation and the provision of adequate responses to the issues raised still has room for improvement. 384 space-group changes were indicated. Other frequently reported problems are unaccounted-for solvent-accessible voids and numerous problems with H atoms.

**Figure 7:**
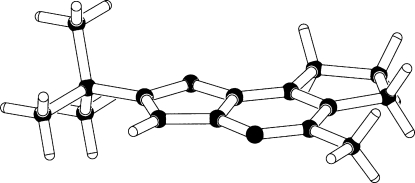
Example of a *P* 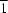 structure at 120 K that has approximate *C*2/*m* space-group symmetry with the molecule on a mirror plane.

Some ALERTS require an in-depth analysis by experts. Investigators not trained in crystallography may have no clue as to what to do with ALERTS about symmetry issues, as may be gleaned from queries such as ‘What does it mean: space group incorrect’. A recent example of a structure with a space-group-related ALERT is the structure report of a small organic molecule that is correctly reported by Portilla *et al.* (2008 ▶) in space group *P* 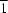 (Fig. 7 ▶). Validation suggests space group *C*2/*m* within default error tolerances as a higher symmetry alternative, which makes sense since the basic molecule has an approximate mirror plane. In fact, this structure easily solves and refines in *C*2/*m* when instructed to do so, although with a higher *R* factor. The evidence against *C*2/*m* is that the atomic displacement parameters in the *t*-butyl moiety are high. In addition, the proposed transformation from triclinic to monoclinic symmetry leads to α and γ angles that differ by 0.3° from the 90° required for monoclinic symmetry. The published structure is based on 120 K data and may well have exact *C*2/*m* symmetry at higher temperature.

The Hirshfeld rigid-bond test (Hirshfeld, 1976 ▶) has proved to be very effective in revealing problems in a structure. It is assumed in this test that two bonded atoms vibrate along the bond with approximately equal amplitude. Significant differences, *i.e.* those which deviate by more than a few standard uncertainties from zero, need close examination. Notorious exceptions are metal-to-carbonyl bonds, which generally show much larger differences (Braga & Koetzle, 1988 ▶).

## 6. What next?

Crystallographic procedures evolve. This also has an impact on structure-validation procedures. A number of currently implemented validation issues are related to data-collection techniques that are based on serial detectors. Those detectors have now largely been superseded by image-plate or CCD-based instruments, which may themselves become obsolete with the arrival of a new generation of (pixel) detectors that allow shutterless data collection. Before the introduction of two-dimensional detectors, corrections for absorption were performed using a multitude of techniques that ranged from purely empirical to an exact calculation based on a description of the crystal shape. Tests were implemented to validate the appropriate use of the chosen method. Nowadays, with two-dimensional detector data, a correction for absorption is mostly of the multi-scan type (*e.g. SADABS*; Sheldrick, 2008 ▶) convoluted with inter-image scaling and optionally preceded by a numerical correction for absorption on the basis of a description of the crystal shape. New up-to-date validation tests for this are needed. Current validation does not yet validate the results of powder diffraction, incommensurate structures and charge-density studies. The same applies to the more involved issues with inorganic compounds. The geometry of a newly determined structure can be validated against similar structures in the CSD (Allen, 2002 ▶; Bruno *et al.*, 2004 ▶). This is easily performed manually but is not easy to automate. An interesting development is the arrival on the market of automated bench-top ‘crystal-to-structure’ instruments. This might pose an interesting challenge to journals and validation software when structure reports from such machines run in black-box mode arrive on editors’ desks. Formal crystallographic training has disappeared in many places, so inexperienced authors might be confronted with difficult to answer ALERT queries. Regular crystallographic training courses are still organized on a national or international basis and should be strongly supported.

## 7. Concluding remarks

Structure validation has become a standard procedure in small-molecule crystallography. It sets a quality standard that is not just based on low final *R* factors and can save a lot of time for both the investigator and the referees of a paper. A short or zero-length list of minor ALERTS may indicate a good structure. Some ALERTS may even point to interesting structural features that would otherwise have gone unnoticed and are worth discussing in a publication. Examples are pseudo-symmetry and short intermolecular contacts. Some ALERTS reveal issues that can only be addressed by experienced crystallographers. An example is whether a given structure is best described as disordered in a centrosymmetric space group or as ordered in a noncentrosymmetric space group (Flack *et al.*, 2006 ▶).

The scope of the currently implemented *checkCIF*/*PLATON* validation procedures is high-resolution small-molecule crystal structures. Extension to large or low-resolution protein structures is not envisioned. As an example, the *PLATON*/*ADDSYM* algorithm that is used to detect missing symmetry requires atomic resolution data.

The automated structure-validation techniques that are currently applied to submissions to *Acta Crystallographica* have essentially eliminated long-standing errors, such as missed higher symmetry, in *Acta Crystallographica Sections B*, *C* and *E*. This is unfortunately not yet the case for many other journals. Class IV structures still appear in the chemical literature. Structures are still published in a too low-symmetry space group despite the many papers on this issue by Dick Marsh entitled ‘*More space group changes*’ (see, for example, Marsh & Herbstein, 1988 ▶). Most major journals state structure validation as a requirement in their Notes for Authors. However, in practice it appears that many structures are published without serious inspection of the crystallographic data by an expert. An often-heard comment is ‘addressing crystallographic details holds up the publication of important chemistry’. In many cases, these crystallographic details are just trivial pieces of information that should already have been included as a standard protocol in the CIF at the end of the structure analysis. Database services, such as the Cambridge Crystallographic Data Centre (CCDC; Allen, 2002 ▶), attempt to sort out some of the obvious problems by consultation with the authors, but the CCDC staff cannot add any judgment or correction without the consent of the authors.
